# The Effect of Blanching on Phytochemical Content and Bioactivity of *Hypochaeris* and *Hyoseris* Species (Asteraceae), Vegetables Traditionally Used in Southern Italy

**DOI:** 10.3390/foods10010032

**Published:** 2020-12-24

**Authors:** Vincenzo Sicari, Monica R. Loizzo, Ana Sanches Silva, Rosa Romeo, Giovanni Spampinato, Rosa Tundis, Mariarosaria Leporini, Carmelo M. Musarella

**Affiliations:** 1 Department of Agraria, “Mediterranea” University of Reggio Calabria, Cittadella Universitaria, Località Feo di Vito, 89122 Reggio Calabria (RC), Italy; vincenzo.sicari@unirc.it (V.S.); rosa.romeo@unirc.it (R.R.); gspampinato@unirc.it (G.S.); carmelo.musarella@unirc.it (C.M.M.); 2 Department of Pharmacy, Health Science and Nutrition, University of Calabria, Via P. Bucci, Edificio Polifunzionale, 87036 Rende (CS), Italy; rosa.tundis@unical.it (R.T.); mariarosarialeporini@tiscali.it (M.L.); 3 National Institute for Agricultural and Veterinary Research (INIAV), I.P., Vairão, 4485-655 Vila do Conde, Portugal; anateress@gmail.com; 4 Center for Study in Animal Science (CECA), ICETA, University of Oporto, 4051-401 Oporto, Portugal

**Keywords:** *Hyoseris radiata*, *Hyoseris taurina*, *Hypochaeris laevigata*, *Hypochaeris radicata*, phytochemicals, antioxidants, obesity, diabetes type 2

## Abstract

The impact of blanching on the phytochemical content and bioactivity of *Hypochaeris laevigata* (HL), *Hypochaeris radicata* (HR), *Hyoseris radiata* (HRA), and *Hyoseris lucida* subsp. *taurina* (HT) leaves was studied and compared to fresh plant materials and residual blanching water. For this purpose, total phenols, flavonoids, carotenoids, and chlorophyll contents were quantified. The antioxidant effect was investigated by using different in vitro tests (β-carotene, ferric reducing ability power (FRAP), 2,2′-Azino-bis(3-ethylbenzothiazoline-6-sulfonic acid) (ABTS) and 2,2-diphenyl-1-picrylhydrazyl (DPPH), whereas the potential inhibitory activity of key enzymes linked to obesity was screened against lipase, α-amylase, and α-glucosidase. Generally, the phytochemical content followed the trend: fresh > blanching water > blanched samples. The same trend was observed in the antioxidant activity independently of the applied test as well as in the inhibition of lipase and carbohydrates-hydrolysing enzymes. In particular, fresh *Hypochaeris laevigata* (HL1) showed the lowest inhibitory concentration 50% (IC_50_) values of 31.3 and 42.7 μg/mL, against α-glucosidase and α-amylase, respectively, whereas fresh *Hyoseris radiata* (HRA1) showed the most promising hypolipidemic activity (IC_50_ value of 39.8 μg/mL). Collectively, these results support the health effect of these wild plants and demonstrated that blanching water should be reused in food preparation since it is a good source of bioactive compounds and its consumption should be recommended in order to increase the uptake of micronutrients.

## 1. Introduction

*Hyoseris* L. and *Hypochaeris* L. species are largely used in Italy not only to prepare salads, omelettes or boiled in soups, but also for medicinal use through the use of infusions using blanching water. *Hyoseris radiata* L. and *Hypochaeris radicata* L. are the most studied species and have several ethnobotanical uses in Italy [1,2,3,4,5,6,7,8]. *Hyoseris lucida* L. subsp. *taurina* (Pamp.) Peruzzi & Vangelisti and *Hypochaeris laevigata* (L.) Ces., Pass. & Gibelli have not been much studied, probably due to their relatively narrow geographical distribution [9,10]. Guarrera and Savo [11,12] reported the use of leaves from *H. radicata* and *H. radiata* as boiled vegetables or salad in Piedmont, Liguria, Marche, Latium, Sardinia, Sicily, and Calabria. In this last region, these species are preserved in olive oil to be consumed as vegetable side dish.

Metabolic syndrome is a cluster of conditions that occur together. These conditions include increased blood pressure, high blood sugar levels, excess body fat around the waist, and abnormal cholesterol or triglyceride levels. [13]. The World Health Organization (WHO) Global Health Observatory estimated through Prophet models that the prevalence of diabetes and obesity in 2030 is likely to increase by 10.1% [14].

Oxidative stress plays a key role in the development of Diabetes Mellitus Type 2 (DMT2) complications [15]. In fact, the metabolic irregularities of diabetes cause an overproduction of superoxide in endothelial cells of both large and small vessels, and, also in the myocardium. Moreover, Găman et al. [16] demonstrated that these irregularities impair tissue glucose uptake and reduces β cell insulin secretion.

The consumption of wild plants represents a key part of the Mediterranean diet, recently recognized by United Nations Educational, Scientific and Cultural Organization UNESCO (UNESCO) as an Intangible Cultural Heritage of Humanity (UNESCO). Although several plants of *Hyoseris* and *Hypochaeris* species have been consumed daily for centuries, there is still no in-depth study on their micronutrient content and health properties after preparation prior to consumption. In the food industry and at a domestic level, blanching is a pretreatment largely used to inactivate enzyme activity, which can affect micronutrient content and preserve vegetables [17]. However, if it is effective in reducing degradation during shelf-life, on the other hand it produces modifications in cell structure and composition with a consequent significant loss of micronutrients in the food matrix [18].

In this context, the evaluation of the impact of blanching on Calabrian traditional vegetables, namely *H. laevigata*, *H. radicata*, *H. radiata,* and *H. lucida* subsp. *taurina,* was investigated. For this purpose, basal leaves were screened for their Total Phenols Content (TPC), Total Flavonoids Content (TFC), chlorophylls and Total Carotenoids Content (TCC) before and after blanching process. β-Carotene bleaching, FRAP, ABTS, and DPPH tests were applied to test the antioxidant activity. The inhibitory effects of enzymes linked to obesity and DMT2 such as lipase, α-glucosidase, and α-amylase were assessed. In order to evaluate the possible loss of bioactive compounds after blanching, the residual blanching water was also investigated.

## 2. Materials and Methods

### 2.1. Chemicals and Reagents

Chemicals and reagents used in this research were purchased from Sigma-Aldrich Chemical Co. Ltd. (Milan, Italy) and VWR International (Milan, Italy). Acarbose from *Actinoplanes* spp. was obtained from Serva (Heidelberg, Germany) whereas β-carotene was from Extrasynthese (Genay-France).

### 2.2. Plant Materials

The collection (Table 1) of the basal rosettes of *H. radicata* and *H. radiata* was carried out at 1157 m a.s.l. in the area of the Aspromonte Massif known as “Cucullaro” (38.171987° N–15.815650° E), a mountain resort in the municipality of Santo Stefano in Aspromonte. The collection of the basal rosettes of *H. lucida* subsp. *taurina* was carried out in the municipality of Scilla (40 m a.s.l., 38.253987° N–15.716493° E), while those of *H. laevigata* at the mountain resort of Trepitò, located at 952 m a.s.l. (38.284906° N–16.046640° E) in the municipality of Molochio. The collection of the basal rosettes was carried out before flower stem development, as traditionally these and similar plants are collected by local people in this way [8,19]. All the collection sites are located in the province of Reggio Calabria (South Italy)

For each of the 4 taxa examined, a minimum quantity of 1000 g of basal rosettes was collected and, afterwards, in the laboratory, a cleaning and separation of the edible leaves was performed manually to obtain a minimum quantity of 200 g of leaves. These were washed several times with distilled water in order to ensure the elimination of any type of residue. Finally, they were placed on absorbent paper and gently dabbed several times, so as to eliminate as much water as possible and thus avoid problems of contamination.

### 2.3. Blanching

To blanche, leaves were placed on a low heat in 1000 mL of hot water (90 °C) for 10 min. The ratio of sample to water was 1:5 (*w*/*w*). After blanching, the samples were cooled to room temperature under running tap water. Finally, the edible parts were drained and the blanching water collected.

### 2.4. Extraction Procedure

Extracts were prepared by mixing 10 g of each sample (fresh and blanched, both ground) with 50 mL of water. The mixture was shaken using a Ultraturrax T-25 (Ika Labortechnik, Janche & Kunkel, Milan, Italy). It was subsequently centrifuged by refrigerated centrifuge Nǜve NF 1200R (Saracalar Kümeevleri, Ankara, Turkey), 10 min at 5000 rpm. Then, the supernatant was filtered through a 0.45 mm Millipore filter (GMF Whatman, Carlo Erba, Milan, Italy) before analysis.

### 2.5. Determination of pH, Titratable Acidity and Total Soluble Solid Content

The pH was determined by direct measurement in a digital potentiometer (Crison Instruments S.A., Milan, Italy). The Total soluble solids (TSS) were measured at 20 °C using a digital Atago Model PR-101 α refractometer (Atago Co. Ltd., Milan, Italy), results were reported as Brix degrees (°Brix). The titratable acidity (TA) was determined using 0.1 N NaOH to pH 8.1. Results were expressed as percentage of monohydrate citric acid.

### 2.6. Total Phenol Content (TPC) and Total Flavonoid Content (TFC)

TPC was determined as described by Sicari et al. [20]. An aliquot of 350 μL of aqueous extract was mixed with Folin-Ciocalteu reagent (1 mL) and 20% Na_2_CO_3_ solution (10 mL). The absorbance was measured at 760 nm in a spectrophotometer (UV-VIS-Agilent 8453) and the results were expressed in mg of gallic acid equivalent (GAE)/100 g fresh weight (FW).

A spectrophotometric method was used to measure the total flavonoid content. TFC was determined using a method based on the formation of a flavonoid-aluminium complex [21]. The extract was mixed with 2% aluminium chloride solution. The samples were incubated at room temperature for 15 min and then measured against a blank at 510 nm. TFC was calculated based on a standard curve and expressed as mg quercetin equivalents (QE)/100 g FW.

### 2.7. Spectrophotometric Determination of Carotenoids

For the carotenoid determination, the spectrophotometric analysis was carried out after extraction [22]. The analyses were carried out in darkness to prevent carotenoid degradation and isomerisation. Before chemical extraction, leaves were homogenised in a blender and an aliquot of 5 g of the sample was weighed into a 50 mL amber coloured flask wrapped with aluminium foil. Then, 100 mL of the solvent mix (hexane/acetone/methanol 2:1:1 *v*/*v*/*v*) was added to the flask and sonicated continuously for 10 min (Misonix Ultrasonic Liquid Processor, Misonix, Inc. 1938, New Highway, Farmingdale, NY, USA).

The extraction was repeated until the sample became colorless. The combined extract was transferred to a separating funnel and 5 mL of distilled water was added to separate polar and nonpolar phases. The nonpolar hexane layer containing carotenoids was collected and concentrated in a rotary evaporator (Heidolph, Schwabach, Germany) until dry. The residue was dissolved in 10 mL of hexane. The total carotenoid content was determined by a spectrophotometric method using a UV-Vis spectrophotometer (Agilent 8453 Technologies, Agilent, Milan, Italy). The absorbance was read at 450 nm. All analyses were performed in triplicate and the results were expressed as mean ± standard deviation (SD).

The results were expressed as g β-carotene/100 g fresh weight of sample.

### 2.8. Chlorophyll Determination

The chlorophyll content was determined spectrophotometrically. The leaves were placed into a mortar and then ground in the dark until the green colour disappeared.

Ground leaf material was extracted with ethanol, filled to 10 mL, centrifuged at 5000 rpm for 3 min and the absorbance of the supernatant solution in a 1 cm cell was read at 440, 649 and 665 nm. Chlorophyll was calculated using the equations previously reported by Lichtenthaler and Buschmann [23].

### 2.9. Radical Scavenging Activity Assays

In ABTS radical scavenging ability test, potassium persulphate solution and ABTS solution were mixed to obtain ABTS radical cation solution [21]. After 12 h, this solution was stabilized (absorbance of 0.70) at 734 nm employing a UV-Vis spectrophotometer (Jenway 6003, Carlo Erba, Milan, Italy). ABTS^+^ solution was mixed with different concentrations of aqueous extracts (from 1 to 400 μg/mL) and the absorbance was read at 734 nm after 6 min.

Another test used to evaluate the radical scavenging activity of our samples is the 2,2-diphenyl-1-picrylhydrazyl (DPPH) assay [21]. The DPPH radical solution was mixed with aqueous extracts (at concentrations in the range from 1 to 1000 μg/mL) and after 30 min the absorbance was read at 517 nm.

### 2.10. Ferric Reducing Ability Power (FRAP) Assay

In the ferric reducing ability power (FRAP) assay, FRAP reagent, tripyridyltriazine (TPTZ), HCl, FeCl_3_, acetate buffer (pH 3.6) were mixed and added to the sample (2.5 mg/mL) [24]. The absorption reaction was read at 595 nm after 30 min of incubation.

### 2.11. β-Carotene Bleaching Test

The ability of samples to inhibit lipid peroxidation was evaluated by using the β-carotene bleaching test [21]. The aqueous extracts (from 2.5 to 100 μg/mL) were mixed with linoleic, Tween 20 acid and β-carotene solution. The absorbance was read at t = 0, and after 30 and 60 min of incubation at 470 nm.

### 2.12. Pancreatic Lipase Inhibitory Activity

The hypolipidemic potential of aqueous extracts was studied by the inhibition of pancreatic lipase [25]. A mixture of lipase (1 mg/mL), 4-nitrophenyl octanoate (substrate), and samples (from 25 to 4000 μg/mL) was prepared. After 30 min of incubation at 37 °C, the Tris-HCl buffer (pH 8.5) was added and the absorbance was measured at 405 nm.

### 2.13. Carbohydrate-Hydrolysing Enzyme Inhibitory Activity

The hypoglycaemic activity of samples was evaluated through inhibition of α-amylase and α-glucosidase enzymes [21]. For α-amylase assay, aqueous extracts (from 25 to 1000 μg/mL) and starch solution were added to the enzyme at room temperature for 5 min, and the absorbance at 540 nm was measured. For α-glucosidase inhibitory activity test, samples extracts (from 25 to 1000 μg/mL) were mixed with enzyme solution, a maltose solution, peroxidase/glucose oxidase (PGO) system-colour reagent solution and *o*-dianisidine (DIAN) solution. This mixture was incubated for 30 min and the absorbance was measured.

### 2.14. Statistical Analysis

All the investigations were performed in triplicate and results were expressed as means of three different experiments ± standard deviation (S.D.). They were processed by analysis of variance (ANOVA). Differences among the samples were analyzed by Turkey’s test using SPSS statistics software (version 17.0, SPSS Inc., Chicago, IL, USA). All the *p* values at < 0.05 were observed as significant.

Principal Component Analysis (PCA) was applied using SPSS software for Windows, version 17.0 (SPSS Inc., Chicago, IL, USA).

## 3. Results and Discussion

In this work the phytochemical content and bioactivity of traditionally consumed wild plants namely *H. laevigata*, *H. radicata*, *H. radiata,* and *H. lucida* subsp. *taurina* were investigated. The edible portions (leaves) were studied, both fresh and after blanching, to assess the impact of processing on these food matrices. Among bioactive phytochemicals, TPC, TFC, lycopene, β-carotene, and chlorophylls were quantified. Samples were studied for their antioxidant potential using different approaches and as inhibitors of enzymes linked to obesity and hyperglycaemia.

### 3.1. Phytochemical Content

Used in the experiment procedures had a significant effect on changes of total soluble solids (TSS), titratable acidity (TA), and pH. The blanching effect of leaves is shown in Table 2. Fresh *Hypochaeris radicata* (HR) and *H. laevigata* (HL) leaves showed a higher TSS contents than the values obtained in the leaves after blanching and in the blanching water. The same trend was observed in the titratable acidity values.

Carotenoid content in raw and cooked leaf samples are shown in Table 3. The reported values show a greater concentration of total carotenoids (expressed in β-Carotene equiv.) in the fresh leaves than in the blanched ones. The blanching water had a significantly higher (*p <* 0.05) carotenoid content than the blanched leaves, but lower than the fresh leaves. A similar trend was found in leaves of both *Hypochaeris* (*leavigata* and *radicata*) and *Hyoseris* (*radiata* and *lucida* subsp. *taurina*). Compared to the initial concentration in the fresh leaves (HL1, HR1, HRA1 and HT1), the lowest carotenoid concentration in blanching water was found in sample HT3, with a value of 205.2 g/100 g, whereas the highest value was found in sample HRA3 (298.1 g/100 g). The presence of carotenoids in blanching water was reported by Parmar et Rupasinghe [26], who observed that an infusion of wild berry stems in hot water had a carotenoid content in the range of 275–417 mg/L. They further showed a high carotenoid content in commercial green tea with average values of 410 and 1332 mg/L for extraction in methanol and hot water respectively. In a study carried out by Loranty et al. [27] on 25 infused fruit and herbal teas, only lutein was present in the infusions, other carotenoids not being found. The highest level of lutein (24.3 mg/200 mL) was found in a Tilias infusion (Edward Tea). Suzuki et Shioii [28] found chlorophyll and carotenoids in seven teas infused from *Camellia sinensis* leaves.

A drastic reduction in chlorophyll was also observed between fresh and blanched samples (from 170.2 to 212.3 for HL1 and HRA1, vs. from 89.3 to 118.4 for HRA2 and HR2, respectively). Blanching reduced the chlorophyll content independently of the species (Table 3).

The concentration of total polyphenols and flavonoids was shown in Table 3. Phenolic compounds are known to be responsible for the antioxidant activity of the food matrix. All fresh samples were characterized by a high TPC content. Leaves of *hypochaeris* (*leavigata* e *radicata*) and *hyoseris* (*radiata* e *lucida* subsp. *taurina*), showed a significantly higher (*p* < 0.05) total polyphenol content in their raw samples compared with their cooked samples [29,30]. The aqueous extract of fresh *H. radicata* showed the highest TPC value of 1103.6 mg GAE/100 g FW followed by *H. lucida* subsp. *taurina* (997.6 mg GAE/100 g FW) (Table 3). Fresh *H. radiata* leaves (HRA1) showed the highest TFC with a value of 855.6 mg QE/100 g FW. It is interesting to note that the blanching process significantly affects both TPC and TFC and that the water in which the leaves are cooked retained a great amount of these bioactive phytochemicals.

The total polyphenol content decreased significantly (*p* < 0.05) after blanching as reported by [31,32,33,34]. In addition, Gawlik-Dziki [35] demonstrated that boiling significantly reduced the polyphenol content of fresh broccoli. Similarly, Sikora et al. [36] reported a significant decrease in total polyphenol and antioxidant components in boiled broccoli. Abu-Ghannam and Jaiswal [37] described a reduction in the total phenolic content of up to 45% at lower blanching temperatures (80–90 °C) within 2 min of blanching and reduction of the total polyphenol content continued within 6 min at high blanching temperatures. Furthermore, as some authors have observed, the degree of leaf fragmentation may be a factor in a greater diffusion of the bioactive compounds from the leaves to the water.

Medina et al. [38] compared infusions obtained from samples at various degrees of fragmentation. Levels of oleuropein were found to be 103 mg/kg in an infusion from a whole sample used as control, and 466 mg/kg in another sample passed through a blender and subsequently an Ultra-Turrax. The greater the degree of leaf fragmentation, the greater the quantities of phenols in the infusions. The total flavonoid content dropped significantly (*p* < 0.05) after boiling. Interestingly, despite heating, the concentration of flavonoids in the blanching water remained high. This may be due to the fact that after boiling there was a greater availability of flavonoids, and a more efficient extraction from the softened cell walls [39].

Moreover, several studies have reported that the increase in bioactive compounds in boiled vegetables may be partly due to the breakdown of cell walls and subcellular structures by boiling, which allows the release of antioxidants [40]. Thus, it is probable that the structural matrix of the cell walls is the factor that determines the cell’s ability to hold onto or breakdown phytochemical compounds.

### 3.2. Antioxidant Activity

The antioxidant activities of *H. laevigata* (HL), *H. radicata* (HR), *H. radiata* (HRA), and *H. lucida* subsp. *taurina* (HT) fresh and blanched leaves were assessed employing in vitro methods: β-carotene bleaching, FRAP, ABTS, and DPPH tests. The resulting blanching water was also screened. To our knowledge this is the first report to evaluate the antioxidant potential of *H. laevigata*, and *H. lucida* subsp. *taurina* leaves, both fresh and processed. Generally, the following antioxidant trend was observed fresh samples > blanching water > blanched samples (Table 4).

A great variability of results was observed in the β-carotene bleaching test. In this assay, the presence of antioxidant compounds minimized the oxidation of β-carotene by hydro-peroxides, which were counteracted by bioactive compounds in the extract. In the present study, both *H. radicata* and *H. laevigata* fresh leaves exerted a greater activity than the other investigated species (IC_50_ = of 46.7 and 54.6 μg/mL after 30 min of incubation, respectively). Blanched samples were less active with a percentage of inhibition of 39.6% and 48.1% at maximum concentration tested (100 μg/mL). A low activity was observed, also with HT1 sample (IC_50_ = 84.7 and 99.1 μg/mL after 30 and 60 min of incubation, respectively).

In FRAP assay, a great ferric reducing power higher than that found for BHT (Butylated Hydroxytoluene) was observed with both HL3 and HL1 samples with values of 92.6 and 84.4 μM Fe(II)/g, respectively (Table 4). A promising ability was observed also with HRA1 (73.5 μM Fe(II)/g).

A different result was observed using ABTS radical cation. In fact, in this test fresh *H. lucida* subsp. *taurina* leaves (HT1) showed a comparable radical scavenging activity to that reported for ascorbic acid (IC_50_ = 1.8 and 1.7 μg/mL, respectively). A promising ABTS radical scavenging potential was noted with the blanching water from the blanching of the leaves of the same species (HT3, IC_50_ = 2.9 μg/mL).

The DPPH free radical scavenging method is based on electron-transfer reaction that produces a violet solution. The DPPH radical is stable at 25 °C. Among the investigated species, aqueous extract of fresh leaves of HR and HL exerted a higher radical scavenging potential (IC_50_ = 18.7 and 24.7 μg/mL, respectively) (Table 4). In addition, interest activity was found for HR3 and HRA3 (IC_50_ = 23.7 and 29.7 μg/mL, respectively).

Previously, Senguttuvan et al. [41] studied the radical scavenging potential of an infusion from the dried leaves of *H. radicata* and found IC_50_ values of 595.23 μg/mL and 2143.1 μmol of Trolox equivalent (TE)/dried weight (DW). Dried leaves are also able to exert their antioxidant activities through other mechanisms, including the protection of lipid peroxidation and ferric reducing power.

Values of 97.99% at 250 mg/mL and 38.69% at 5 mg/mL were recorded by Senguttuvan et al. [41] for Indian *H. radicata* dried leaves in DPPH and ABTS test, respectively. Successively the same research group showed that the oral administration of *H. radicata* methanolic leaf and root extracts and isolated compounds proved to be significant, promising candidates able to quench free radicals. The antioxidant effect was more pronounced in the animals treated with root extract, probably due its high content in alkaloids, flavonoids, saponins and terpenoids that could act as antioxidant compounds [41]. Lower antioxidant activity was recorded for *H. radicata* collected in Latium [42] where IC_50_ average values of 2.02 and 2.33 mg/mL were found for fresh and boiled leaves, respectively in DPPH assay. Average values of 6.2 and 2.3 mmol/kg FW were found for fresh and boiled leaves in ABTS test, respectively.

Compared to the Calabria sample HRA1, a lower ferric reducing power was recorded for *H. radiata* fresh leaves collected in Liguria that showed FRAP value of 31.1 mM Fe (II) /Kg. More recently, Souilah et al. [43] investigated the antioxidant effect of *n*-butanol, dichloromethane and ethyl acetate fractions of the aerial parts of *Hypochaeris laevigata* var. *hipponensis*. The highest DPPH radical scavenging activity was exhibited by *n*-Butanol extract (IC_50_ = 8.12 μg/mL) followed by ethyl acetate extract (IC_50_ = 8.70 μg/mL). This last extract was also able to exert a potent ABTS radical cation inactivation (IC_50_ = 4.32 μg/mL). The following rank dichloromethane, *n*-butanol and ethyl acetate in protection of lipid peroxidation was found.

### 3.3. Inhibition of Enzymes linked to Obesity

The study of bioactive foods useful in the prevention and management of metabolic diseases including obesity and diabetes is a topic of great interest for researchers in the area of food science. To our knowledge, no previous studies have investigated the species *Hypochaeris* and *Hyoseris* for their ability to inhibit carbohydrate-hydrolyzing and lipase enzymes. All investigated samples exerted inhibitory activity on enzymes linked to obesity and DMT2 in a concentration dependent manner (Table 5).

Fresh *H. laevigata* leaves (HL1) exerted a promising α-amylase inhibitory activity with IC_50_ value of 42.7 μg/mL which is lower compared to positive control acarbose (IC_50_ = 50.1 μg/mL). A notable activity was observed, also in the blanching water (HL5, IC_50_ = 56.8 μg/mL). The HL1 sample showed the greatest α-glucosidase inhibitory activity followed by *H. radicata* (HR1) with IC_50_ values of 31.3 and 37.6 μg/mL, respectively. Both results are comparable with those found for acarbose (IC_50_ = 35.5 μg/mL). Also, in this case, blanching significantly affected the bioactivity of the samples. Fresh *H. radiata* leaves (HRA1) showed the highest lipase inhibitory activity (IC_50_ = 39.8 μg/mL) whereas, values of 52.4 and 58.2 μg/mL were found for HR1 and HL1, respectively. Unlike what happened for the carbohydrate hydrolysing enzymes, the blanching water has only a minimal inhibitory activity (IC_50_ values ranged from 62.8 to 78.8 for HR3 and HT3, respectively) testifying that compounds able to inhibit lipase enzyme are retained in the matrix also after blanching.

Among our investigated samples, only *H. radicata* had been previously investigated for its hypoglycaemic activity. However, our data disagree with Ko et al. [44], who did not find α-glucosidase inhibitory activity at the maximum concentration tested of 1000 μg/mL. Probably, this lack of activity was due the relatively low content of TPC and TFC found by the authors.

### 3.4. Principal Component Analysis (PCA)

PCA was performed to identify accession groups and to determine the axes and the characters significantly contributing to the variation. In this procedure, the similarity matrix was used to generate eigenvalues and scores for the accessions. The first two principal components, which accounted for the highest variation, were then used to plot two-dimensional scatter plots [45].

**Figure 1:**
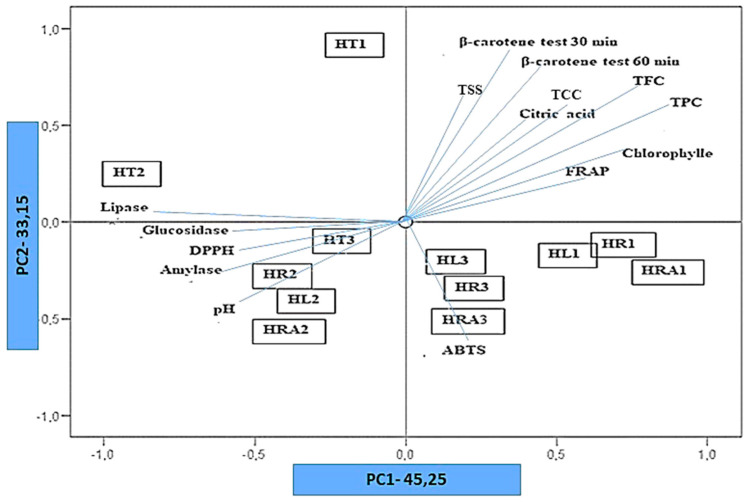
Factor loadings for principal components (PC) PC1 and PC2 and scatter plot of all. samples for principal component analysis.

PCA was applied to differentiate the four different taxa of *Hypochaeris* and *Hyoseris.* By choosing eigenvalues greater than one (>1), the dimensionality was reduced from 16 variables to two principal components (PC). PCA results revealed that the first two principal components explained total variance completely 78.4%. The loadings of first and second principal components (PC1 and PC2) accounted for 45.25 and 33.15% of the variance, respectively (Figure 1). The first component (PC1) is highly positively correlated with Chlorophylls, and FRAP. The second component (PC2) is positively correlated with TSS, β-carotene bleaching test t = 30 min, β-carotene bleaching test t = 60 min, while citric acid, β-Carotene, TPC, and TFC) are positively correlated with component 1 and component 2. pH, DPPH, α-glucosidase, and α-amylase show a negative correlation for PC1 and PC2.

The bi-dimensional PCA analysis clearly classifies the similarities or differences of the botanic species and the treatments performed. The score plot analysis clearly classifies the species HL1, HR1, HRA1, and HR3, HL3, HRA3 in the lower right region of the PCA score plot. This shows that fresh plants and blanching water maintain a higher bioactivity than blanched leaves.

## 4. Conclusions

The present study assessed for the first time the impact of the blanching process on the phytochemical content and bioactivity of the spontaneous plants namely *Hypochaeris laevigata*, *H. radicata*, *Hyoseris radiata,* and *H. lucida* subsp. *taurina.* Traditionally, these species are widely consumed in Central and South Italy, both fresh and after blanching. For this purpose, fresh and blanched samples as well as residual blanching water were studied. The blanching process determined a reduction in the content of all investigated phytochemical classes. At the same time, the analysis of the data showed that the blanching water retains most of the bioactive compounds and for this reason it is characterized by a good antioxidant and inhibitory activity against enzymes linked to obesity and related diseases such as diabetes type 2. For this reason, the consumption of the fresh spontaneous plant and the reutilization of residual blanching water should be promoted in order to ensure the right amounts of healthy micronutrients able to counteract oxidative stress and related diseases.

## Figures and Tables

**Table 1:** Sampling locations.

Sampling Locations	Latitude	Longitude	Altitude m a.s.l.
Cucullaro	38.166667°	15.850000°	1300
Scilla	38.250000°	15.733333°	73
Trepitò	38.316667°	16.150000°	880

**Table 2:** Total soluble solid content (TSS), Titratable acidity (TA), and pH of *Hypochaeris* and *Hyoseris* extracts.

Sample	TSS	TA	pH
***Hypochaeris leavigata (HL)***			
HL1	0.6 ± 0.05 ^a^	5.2 ± 2.0 ^a^	6.0 ± 2.6 ^b^
HL2	0.1 ± 0.01 ^c^	0.8 ± 0.07 ^c^	6.6 ± 2.7 ^a^
HL3	0.4 ± 0.04 ^b^	1.9 ± 1.3 ^b^	6.2 ± 2.4 ^b^
**Sign.**	**	**	**
***Hypochaeris radicata (HR)***			
HR1	0.6 ± 0.07 ^a^	4.5 ± 1.9 ^a^	6.0 ± 2.6 ^b^
HR2	0.2 ± 0.02 ^b^	0.7 ± 0.06 ^c^	6.4 ± 2.3 ^a^
HR3	0.7 ± 0.08 ^a^	2.1 ± 1.4 ^b^	6.5 ± 2.5 ^a^
**Sign.**	**	**	**
***Hyoseris radiata*** **(HRA)**			
HRA1	0.4 ± 0.03 ^a^	6.8 ± 2.9 ^a^	5.8 ± 2.2 ^b^
HRA2	0.3 ± 0.02 ^b^	1.4 ± 1.1 ^c^	6.4 ± 2.4 ^a^
HRA3	0.4 ± 0.04 ^a^	1.7 ± 1.2 ^b^	6.2 ± 2.3 ^a^
**Sign.**	**	**	**
***Hyoseris lucida*** **subsp. *taurina* (HT)**			
HT1	0.9 ± 0.1 ^a^	6.3 ± 2.7 ^a^	5.9 ± 2.2 ^b^
HT2	0.7 ± 0.09 ^b^	2.3 ± 1.5 ^c^	6.2 ± 2.4 ^a^
HT3	0.5 ± 0.06 ^a^	2.1 ± 1.2 ^b^	6.4 ± 2.5 ^a^
**Sign.**	**	**	**

Data are reported to mean ± Standard Deviation (SD) (*n* = 3). 1: Extract fresh plant materials; 2: Extract plant materials after blanching; 3: Blanching water extract. ** Significance at *p* < 0.01. Results followed by different letters in a same column are significantly different (*p* < 0.05) by Tukey’s multiple. range test.

**Table 3:** Phytochemical content of *Hypochaeris* and *Hyoseris* extracts.

Sample	TCC (mg βC/100 g FW)	Chlorophylls (mg/kg FW)	TPC (mg GAE/100 g FW)	TFC (mg QE/100 g FW)
***Hypochaeris leavigata (HL)***				
HL1	351.8 ± 5.1 ^a^	170.2 ± 3.2 ^a^	946.5 ± 6.2 ^a^	732.7 ± 6.0 ^a^
HL2	215.9 ± 4.7 ^c^	106.8 ± 3.1 ^c^	378.1 ± 5.1 ^c^	179.4 ± 3.2 ^b^
HL3	298.1 ± 4.9 ^b^	163.0 ± 3.2 ^b^	623.0 ± 5.9 ^b^	569.6 ± 5.7 ^c^
**Sign**	**	**	**	**
***Hypochaeris radicata (HR)***				
HR1	449.5 ± 5.5 ^a^	191.7 ± 3.6 ^a^	1103.6 ± 6.5 ^a^	769.2 ± 6.1 ^a^
HR2	236.9 ± 4.5 ^c^	118.4 ± 3.3 ^c^	434.3 ± 5.4 ^c^	322.5 ± 4.2 ^c^
HR3	266.0 ± 4.6 ^b^	143.2 ± 3.4 ^b^	550.8 ± 5.6 ^b^	375.0 ± 4.3 ^b^
**Sign**	**	**	**	**
***Hyoseris radiata*** **(HRA)**				
HRA1	386.7 ± 5.2 ^a^	212.3 ± 4.0 ^a^	975.3 ± 6.2 ^a^	855.6 ± 5.9 ^a^
HRA2	181.3 ± 3.5 ^c^	89.3 ± 2.9 ^c^	380.1 ± 4.2 ^c^	277.1 ± 3.8 ^c^
HRA3	240.6 ± 4.5 ^b^	136.7 ± 3.2 ^b^	550.7 ± 5.2 ^b^	370.4 ± 4.0 ^b^
**Sign**	**	**	**	**
***Hyoseris lucida*** **subsp. *taurina* (HT)**				
HT1	532.4 ± 5.8 ^a^	187.4 ± 3.4 ^a^	997.6 ± 6.3 ^a^	796.8 ± 6.0 ^a^
HT2	202.6 ± 3.5 ^b^	106.4 ± 3.1 ^c^	416.1 ± 5.3 ^c^	436.2 ± 5.3 ^b^
HT3	205.2 ± 3.6 ^b^	109.4 ± 3.2 ^b^	627.5 ± 5.8 ^b^	447.3 ± 5.4 ^b^
**Sign.**	**	**	**	**

Data are reported to mean ± Standard Deviation (SD) (*n* = 3). Data are expressed as mean ± S.D. (*n* = 3). 1: Extract fresh plant materials; 2: Extract plant materials after blanching; 3: Blanching water extract. ** Significance at *p* < 0.01. Results followed by different letters in a same column are significantly different (*p* < 0.05) by Tukey’s multiple range test.

**Table 4:** Antioxidant activity of *Hypochaeris* and *Hyoseris* extracts.

Samples	β-Carotene Bleaching Test IC_50_ (µg/mL)		FRAP μMFe (II)/g	ABTS IC_50_ (µg/mL)	DPPH IC_50_ (µg/mL)
	t = 30 min	t = 60 min			
***Hypochaeris leavigata*** **(HL)**					
HL1	46.7 ± 3.0 ^b^	48.5 ± 3.1 ^b^	84.4 ± 3.8 ^b^	4.7 ± 0.8 ^c^	24.7 ± 2.2 ^c^
HL2	39.6 ± 2.3 ^c^	30.1 ± 1.9 ^c^	39.1 ± 3.4 ^c^	7.9 ± 1.2 ^a^	43.6 ± 3.5 ^a^
HL3	48.7 ± 3.1 ^a^	59.0 ± 3.6 ^a^	92.6 ± 4.0 ^a^	5.9 ± 0.9 ^b^	37.6 ± 3.1 ^b^
**Sign.**	**	**	**	**	**
***Hypochaeris radicata*** **(HR)**					
HR1	54.6 ± 3.3 ^b^	58.5 ± 3.4 ^b^	41.6 ± 3.2 ^c^	7.8 ± 1.2 ^c^	18.7 ± 1.5 ^c^
HR2	48.1 ± 2.8 ^c^	35.7 ± 1.4 ^c^	53.8 ± 3.5 ^a^	12.3 ± 1.5 ^a^	41.6 ± 3.0 ^a^
HR3	60.5 ± 3.8 ^a^	63.9 ± 3.8 ^a^	49.2 ± 3.5 ^b^	13.7 ± 1.6 ^b^	23.7 ± 1.9 ^b^
**Sign.**	**	**	**	**	**
***Hyoseris radiata*** **(HRA)**					
HRA1	51.7 ± 3.5 ^a^	41.7 ± 2.4 ^a^	73.5 ± 4.1 ^a^	8.8 ± 0.9 ^c^	37.6 ± 2.2 ^b^
HRA2	18.7 ± 1.3 ^c^	14.1 ± 0.8 ^c^	38.9 ± 3.1 ^c^	11.8 ± 1.1 ^a^	51.5 ± 3.2 ^a^
HRA3	26.4 ± 1.6 ^b^	29.2 ± 1.1 ^b^	41.1 ± 2.9 ^b^	10.1 ± 1.0 ^b^	29.7 ± 2.0 ^c^
**Sign.**	**	**	**	**	**
***Hyoseris lucida* subsp. *taurina* (HT)**					
HT1	84.7 ± 3.9 ^a^	99.1 ± 4.2 ^a^	54.7 ± 3.6 ^a^	1.8 ± 0.3 ^c^	33.6 ± 2.0 ^c^
HT2	41.4 ± 2.1 ^b^	23.8 ± 1.8 ^c^	41.0 ± 3.1 ^c^	4.1 ± 0.9 ^a^	54.5 ± 3.2 ^a^
HT3	39.1 ± 2.5 ^c^	31.1 ± 2.1 ^b^	53.2 ± 3.5 ^b^	2.9 ± 0.5 ^b^	42.6 ± 2.5 ^b^
**Sign.**	**	**	**	**	**

Data are expressed as mean ± Standard Deviation (SD) (*n* = 3). 1: Extract fresh plant materials; 2: Extract plant materials after blanching; 3: Blanching water extract. Ferric Reducing Antioxidant Power (FRAP); Antioxidant Capacity Determined by Radical Cation (ABTS+); DPPH Radical Scavenging Activity Assay. ^a^: [100 μg/mL]. Propyl gallate (IC_50_ = 0.09 ± 0.04 μg/mL after t = 30 min and t= 60 min of incubation) was used as control positive in β-carotene bleaching test, BHT (IC_50_ = 63.2 ± 2.3 μMFe (II)/g) in FRAP assay and ascorbic acid in ABTS and DPPH radical scavenging test (IC_50_ = 5.0 ± 0.8 and 1.7 ± 0.1 μg/mL, respectively). One-way ANOVA followed by Tukey’s multiple range test was applied for statistical analysis. Different letters in the same column are significantly different ** at *p* < 0.01.

**Table 5:** Lipase, α-amylase, and α-glucosidase inhibitory activity [IC_50_ (μg/mL)] of *Hypochaeris* and *Hyoseris* extracts.

Sample	Lipase	α-Amylase	α-Glucosidase
***Hypochaeris leavigata* (HL)**			
HL1	58.2 ± 1.5 ^c^	42.7 ± 1.3 ^c^	31.3 ± 1.3 ^c^
HL2	85.0 ± 1.8 ^a^	94.9 ± 2.0 ^a^	81.6 ± 1.8 ^a^
HL3	66.4 ± 1.7 ^b^	56.8 ± 1.5 ^b^	62.3 ± 1.6 ^b^
Sign.	**	**	**
***Hypochaeris radicata* (HR)**			
HR1	52.4 ± 1.5 ^c^	56.9 ± 1.4 ^c^	37.6 ± 1.3 ^c^
HR2	81.4 ± 1.7 ^a^	112.9 ± 2.1 ^a^	118.8 ± 2.3 ^a^
HR3	62.8 ± 1.7 ^b^	90.1 ± 2.0 ^b^	43.9 ± 1.4 ^b^
Sign.	**	**	**
***Hyoseris radiata* (HRA)**			
HRA1	39.8 ± 1.4 ^c^	79.4 ± 1.7 ^c^	41.9 ± 1.4 ^c^
HRA2	85.7 ± 1.8 ^a^	99.1 ± 1.9 ^a^	84.5 ± 1.8 ^a^
HRA3	65.7 ± 1.7 ^b^	83.4 ± 1.8 ^b^	51.9 ± 1.5 ^b^
Sign.	**	**	**
***Hyoseris lucida*** **subsp. *taurina* (HT)**			
HT1	73.5 ± 1.7 ^c^	74.0 ± 1.7 ^c^	63.1 ± 1.6 ^c^
HT2	89.6 ± 1.9 ^a^	94.2 ± 1.9 ^a^	94.9 ± 1.9 ^a^
HT3	78.8 ± 1.8 ^b^	82.8 ± 1.6 ^b^	74.4 ± 1.7 ^b^
Sign.	**	**	**

Data are expressed to mean ± Standard Deviation (SD) (*n* = 3). 1: Extract fresh plant materials; 2: Extract plant materials after blanching; 3: Blanching water extract. Orlistat used as positive control in lipase test (IC_50_ = 37.4 ± 1.0). Acarbose used as positive control in α-amylase and α-glucosidase tests (IC_50_ = 50.1 ± 1.3 and 35.5 ± 0.9 respectively for α-amylase α-glucosidase). One-way ANOVA followed by Tukey’s multiple range test was applied for statistical analysis. Different letters in the same column are significantly different ** at *p* < 0.01.

## References

[1] Gastaldo P., Barberis G., Fossati F. (1978). Le piante della medicina tradizionale nei dintorni di Praglia (Appennino Ligure-Piemontese). Atti. Dell’accad. Ligur. Sci. Lett..

[2] Bellomaria B., Lattanzi E. (1982). Le piante del territorio di Cupra Marittima (Marche) attualmente usate nella medicina popolare. Arch. Bot. Biogeogr. Ital..

[3] Guarrera P.M. (1990). Usi tradizionali delle piante in alcune aree marchigiane. Inf. Bot. Ital..

[4] Ghirardini M.P., Carli M., Del Vecchio N., Rovati A., Cova O., Valigi F., Agnetti G., Macconi M., Adamo D., Traina M. (2007). The importance of a taste. A comparative study on wild food plant consumption in twenty-one local communities in Italy. J. Ethnobiol. Ethnomed..

[5] Cornara L., La Rocca A., Marsili S., Mariotti M.G. (2009). Traditional uses of plants in the Eastern Riviera (Liguria, Italy). J. Ethnopharmacol..

[6] Lentini F., Venza F. (2007). Wild food plants of popular use in Sicily. J. Ethnobiol. Ethnomed..

[7] Giambanelli E., D’antuono L., Ferioli F., Frenich A., Romero-González R. (2018). Sesquiterpene lactones and inositol 4-hydroxyphenylacetic acid derivatives in wild edible leafy vegetables from Central Italy. J. Food Comp. Anal..

[8] Maruca G., Spampinato G., Turiano D., Laghetti G., Musarella C.M. (2019). Ethnobotanical notes about medicinal and useful plants of the Reventino Massif tradition (Calabria region, Southern Italy). Genet. Resour. Crop. Evol..

[9] Brullo S., Minissale P., Siracusa G., Spampinato G. (1990). Considerazioni fitogeografiche su *Hyoseris taurina* (Pamp.) Martinoli (Asteraceae). Giorn. Bot. Ital..

[10] Pignatti S., Guarino R., La Rosa M. (2018). Flora d’Italia.

[11] Guarrera P.M., Savo V. (2013). Perceived health properties of wild and cultivated food plants in local and popular traditions of Italy: A review. J. Ethnopharmacol..

[12] Guarrera P.M., Savo V. (2016). Wild food plants used in traditional vegetable mixtures in Italy. J. Ethnopharmacol..

[13] Furukawa S., Fujita T., Shimabukuro M., Iwaki M., Yamada Y., Nakajima Y., Nakayama O., Makishima M., Matsuda M., Shimomura I. (2004). Increased oxidative stress in obesity and its impact on metabolic syndrome. J. Clin. Investig..

[14] Ampofo A.G., Boateng E.B. (2020). Beyond 2020: Modelling obesity and diabetes prevalence. Diabetes Res. Clin. Pract..

[15] Giacco F., Brownlee M. (2010). Oxidative stress and diabetic complications. Circ. Res..

[16] Găman M., Epingeac M., Diaconu C., Găman A. (2019). Oxidative stress levels are increased in type 2 diabetes mellitus and obesity. J. Hypertens..

[17] Lucci P., Pacetti D., Loizzo M.R., Frega N.G., Jaiswal Amit K. (2016). Canning: Impact on food products quality attributes. Food Processing Technologies, Impact on Product Attributes.

[18] Lin C.H., Chang C.Y. (2005). Textural change and antioxidant properties of broccoli under different cooking treatments. Food Chem..

[19] Musarella C.M., Paglianiti I., Cano-Ortiz A., Spampinato G. (2019). Indagine etnobotanica nel territorio del Poro e delle Preserre Calabresi (Vibo Valentia, S-Italia). Atti. Soc. Tosc. Sci. Nat. Mem. Ser. B.

[20] Sicari V., Loizzo M.R., Tundis R., Mincione A. (2018). Pellicanò, *Portulaca oleracea* L. (Purslane) extracts display antioxidant and hypoglycaemic effects. J. Appl. Bot. Food Qual..

[21] Loizzo M.R., Tundis R., Sut S., Dall’acqua S., Ilardi V., Leporini M., Falco T., Sicari V., Bruno M. (2020). High-Performance Liquid Chromatography/Electrospray Ionization Tandem Mass Spectrometry (HPLC-ESI-MSn) analysis and bioactivity useful for prevention of “diabesity” of *Allium commutatum* Guss. Plant Foods Hum. Nutr..

[22] Fish W.W., Perkins-Veazie P., Collins J.K. (2002). A quantitative assay for lycopene that utilizes reduced volumes of organic solvents. J. Food Compos. Anal..

[23] Lichtenthaler H.K., Buschmann C. (2001). Current Protocols in Food Analytical Chemistry.

[24] Loizzo M.R., Sicari V., Pellicanò T., Xiao J., Poiana M., Tundis R. (2019). Comparative analysis of chemical composition, antioxidant and anti-proliferative activities of Italian *Vitis vinifera* by-products for a sustainable agro-industry. Food Chem. Toxicol..

[25] El-Shiekh R.A., Al-Mahdy D.A., Hifnawy M.S., Abdel-Sattar E.A. (2019). *In-vitro* screening of selected traditional medicinal plants for their anti-obesity and anti-oxidant activities. S. Afr. J. Bot..

[26] Parmar I., Vasantha Rupasinghe H.P. (2015). Antioxidant Capacity and Anti-diabetic Activity of Wild Berry Stem Infusions. Eur. J. Med. Plant..

[27] Loranty A., Rembiałkowska E., Rosa E.A.S., Bennet R.S. (2010). Identification, quantification and availability of carotenoids and chlorophylls in fruit, herb and medicinal teas. J. Food Comp. Anal..

[28] Suzuki Y., Shioi Y. (2003). Identification of chlorophylls and carotenoids in major teas by high-performance liquid chromatography with photodiode array detection. J. Agric. Food Chem..

[29] Kao F.J., Chiu Y.S., Chiang W.D. (2014). Effect of water cooking on the antioxidant capacity of carotenoid-rich vegetables in Taiwan. J. Food Drug Anal..

[30] Prasanna K.D., Gunathilake P., Somathilaka Ranaweera K.K.D., Vasantha Rupasinghe H.P. (2018). Effect of Different Cooking Methods on Polyphenols, Carotenoids and Antioxidant Activities of Selected Edible Leaves. Antioxidants.

[31] Amin I., Zamaliah M., Marjan C., Foong W. (2004). Total antioxidant activity and phenolic content in selected vegetables. Food Chem..

[32] Amin I., Lee W.L. (2005). Effect of different blanching times on antioxidant properties in selected cruciferous vegetables. J. Sci. Food Agric..

[33] Bamidele O.P., Fasogbon M.B., Adebowale O.J., Adeyanju A.A. (2017). Effect of Blanching Time on Total Phenolic, Antioxidant Activities and Mineral Content of Selected Green Leafy Vegetables. Curr. J. Appl. Sci. Technol..

[34] Hong-Wei X., Zhongli P., Li-Zhen D., El-Mashad H.M., Xu-Hai Y., Mujumdar A.S., Zhen-Jiang G., Qian Z. (2017). Recent developments and trends in thermal blanching—A comprehensive review. Inf. Process. Agric..

[35] Gawlik-Dziki U. (2008). Effect of hydrothermal treatment on the antioxidant properties of broccoli (*Brassica oleracea* var. botrytis italica) florets. Food Chem..

[36] Sikora E., Cieslik E., Leszczynska T., Filipiak-Florkiewicz A., Pisulewski P.M. (2008). The antioxidant activity of selected cruciferous vegetables subjected to aquathermal processing. Food Chem..

[37] Jaiswal A.K., Abu-Ghannam N., Amit J. (2015). Blanching as a treatment process: Effect on polyphenols and antioxidant capacity of cabbage. Processing and Impact on Active Components in Food.

[38] Medina E., Romero C., García P., Brenes M. (2019). Characterization of bioactive compounds in commercial olive leaf extracts, and olive leaves and their infusions. Food Funct..

[39] Wachtel-Galor S., Wong K.W., Benzie I.F. (2008). The effect of cooking on Brassica vegetables. Food Chem..

[40] Yamaguchi T., Katsuda M., Oda Y., Terao J., Kanazawa K., Oshima S., Inakuma T., Ishiguro Y., Takamura H., Matoba T. (2003). Influence of polyphenol and ascorbateoxidases during cooking process on the radical-scavenging activity of vegetables. Food Sci. Technol. Res..

[41] Senguttuvan J., Paulsamy S., Karthika K. (2014). Phytochemical analysis and evaluation of leaf and root parts of the medicinal herb, *Hypochaeris radicata* L. for *in vitro* antioxidant activities. Asian Pac. J. Trop. Biomed..

[42] Savo V., Salomone F., Mattoni E., Tofani D., Caneva G. (2019). Traditional salads and soups with wild plants as a source of antioxidants: A comparative chemical analysis of five species growing in central Italy. Evid. Based Compl. Alter. Med..

[43] Souilah N., Ullah Z., Bendif H., Medjroubi K., Hazmoune T., Hamel T., Öztürk M., Nieto G., Akkal S. (2020). Phenolic compounds from an algerian endemic species of *Hypochaeris laevigata* var. *hipponensis* and investigation of antioxidant activities. Plants.

[44] Ko Y.M., Eom T.K., Song S.K., Jo G.Y., Kim J.S. (2017). Tyrosinase and α-Glucosidase Inhibitory Activities and Antioxidant Effects of Extracts from Different Parts of *Hypochaeris radicata*. Korean J. Med. Crop. Sci..

[45] D’agostino M.F., Sanz J., Martínez-Castro I., Giuffrè A.M., Sicari V., Soria A.C. (2014). Statistical analysis for improving data precision in the SPME GC–MS analysis of blackberry (*Rubus ulmifolius* Schott) volatiles. Talanta.

